# Dibromoacetic Acid Induced Hepatotoxicity in Mice through Oxidative Stress and Toll-Like Receptor 4 Signaling Pathway Activation

**DOI:** 10.1155/2019/5637235

**Published:** 2019-11-20

**Authors:** Tingting Gong, Wenbo Jiang, Zhijian Gao, Yingying Chen, Shuying Gao

**Affiliations:** Department of Toxicological Science, School of Public Health, Harbin Medical University, Harbin 150081, China

## Abstract

Dibromoacetic acid (DBA) is one of haloacetic acids, often as a by-product of disinfection in drinking water. DBA is a multiple-organ carcinogen in rodent animals, but little research on its hepatotoxicity has been conducted and its mechanism has not been elucidated. In this study, we found that DBA could induce obvious hepatotoxcity in Balb/c mice as indicated by histological changes, increasing serum level of alanine aminotransferase (ALT) and aspartate aminotransferase (AST), and accumulation of hepatic glycogen, after the mice were administered DBA at doses of 1.25, 5, and 20 mg/kg body weight for 28 days via oral gavage. In mechanism study, DBA induced oxidative stress as evidenced by increasing the level of malondialdehyde (MDA), reactive oxygen species (ROS) in the liver, advanced oxidative protein products (AOPPs) in the serum, and decreasing the level of glutathione (GSH) in the liver. DBA induced inflammation in the liver of the mice which is supported by increasing the production of tumor necrosis factor-*α* (TNF-*α*) and the mRNA levels of TNF-*α*, interleukin-6 (IL-6), interleukin-1*β* (IL-1*β*), and nuclear factor *κ*B (NF-*κ*B) in the liver. DBA also upregulated the protein levels of Toll-like receptor (TLR) 4, myeloid differentiation factor 88 (MyD88), tumor necrosis factor receptor-associated factor 6 (TRAF6), inhibitor of nuclear factor *κ*B alpha (I*κ*B-*α*), nuclear factor *κ*B p65 (NF-*κ*B p65), and the phosphoralation of P38 mitogen-activated protein kinase (P38MAPK) and c-Jun N-terminal kinase (JNK). *Conclusion*. DBA could induce hepatotoxicity in mice by oral exposure; the mechanism is related to oxidative stress, inflammation, and Toll-like receptor 4 signaling pathway activation.

## 1. Introduction

Dibromoacetic acid (DBA) is a disinfection by-product commonly found in finished drinking water as a result of chlorination/oxidation processes. It was nominated to the National Toxicology Program by the United States Environmental Protection Agency for toxicity and carcinogenicity studies [1]. Some rodent studies have demonstrated many effects including reproductive and developmental toxicity, neurotoxicity, and immunotoxicity on mice and rats [2–5]. The National Institute of Environmental Health Sciences (NIEHS) determined that DBA is a multiple-organ (including liver and lung) carcinogen in rats and mice following prolonged exposure via drinking water and a significant increase was observed in the incidence of hepatocellular tumors in 2-year studies at exposures as low as 50 mg/l [6]. The liver is not only a detoxification organ but an immune organ, playing vital functions in the maintenance and performance of the body. During the detoxification process of various xenobiotic chemicals, the reactive chemical intermediates damage the liver causing hepatotoxicity. Liver injury associated with devastating consequences can be caused by many chemicals. However, few studies on the hepatotoxicity of DBA were identified and the mechanism is not clear.

Many chemicals and drugs are oxidized by cytochrome P450 (CYP450) enzymes to N-acetyl-*p*-benzoquinone imine (NAPQI), a highly reactive intermediate, which is detoxified by covalent binding with glutathione (GSH). Excessive NAPQI depletes hepatic GSH pool and causes oxidative stress and hepatocellular death [7]. DBA is biotransformed to glyoxylate primarily in the liver and catalyzed by glutathione-*S* transferase zeta (GST*ζ*) by a GSH-dependent process [8].

Toll-like receptors (TLRs) are not only important in regulating innate and adaptive immune response but also associated with inflammatory liver diseases [9]. TLRs are a family of transmembrane proteins that represent the major pattern recognition receptors (PRRs) which are expressed on Kupffer cells as well as other cell type in the liver [10]. Among TLRs, TLR-4 is a crucial mediator of inflammation and innate immune activation, containing the myeloid differentiation factor 88- (MyD88-) dependent pathway and MyD88-independent pathway [11]. The MyD88-dependent pathway can lead to the activation of nuclear factor *κ*B (NF-*κ*B) and trigger inflammatory responses exacerbating the liver injury [12]. And once NF-*κ*B is activated in cytoplasm via I*κ*B-*α* kinase (IKK) phosphorylation and degradation of I*κ*B-*α*, it will shift to nuclei and binding with DNA consensus sequence to induce inflammation-related transcription process such as tumor necrosis factor-*α* (TNF-*α*), interleukin-6 (IL-6), and interleukin-1*β* (IL-1*β*) [13, 14].

Therefore, in the present study, we aim to explore whether DBA could induce hepatotoxicity in vivo and further search the underlying mechanisms related to oxidative damage and TLR4 pathway activation.

## 2. Materials and Methods

### 2.1. Chemicals and Reagent

Dibromoacetic acid (MW: 217.84; CAS: 631-64-1; purity 99%) was obtained from Sigma-Aldrich (St. Louis, MO, USA). DBA solution was prepared by dissolving the compound in deionized water and adjusting pH to 6.5 with 1 N NaOH.

### 2.2. Animal Studies

Six- to eight-week-old Balb/c mice (*n* = 40) were purchased from Harbin Medical University Laboratory Animal Center (Harbin, China). The male and female mice were separately housed in specific pathogen-free facilities maintained at 22 ± 3°C with a 40-70% relative humidity and a 12 h light : dark cycle and had ad libitum access to standard rodent chow and filtered water. After acclimation for a week, the mice were assigned randomly into four groups by weight (*n* = 5/gender/dose group). Mice were administered with deionized water (vehicle control) or DBA (1.25, 5, or 20 mg/kg body weight) solution by daily gavage (at the volume of 10 ml/kg) for consecutive 28 days. Body weight was measured and recorded every 7 days. The weights of the livers were measured when the mice were sacrificed, and the relative weights of the liver of each mouse were calculated by the formula of liver weight/body weight∗100%. All procedures in this study were approved by Harbin Medical University Ethics Committee for animal research and conformed to the Guide for the Care and Use of Laboratory Animals prepared by the National Academy of Sciences and published by the National Institutes of Health.

### 2.3. Blood Collection and Liver Homogenates Preparation

At 24 h after the final dosing, each mouse was euthanized by cervical dislocation. The serum was separated from whole blood, aliquoted into Eppendorf tubes, and frozen at −80°C until used in analyses. The livers were aseptically removed and snap-frozen in liquid nitrogen. A 10% homogenate was prepared in 50 mM phosphate buffer (pH 7) using a polytron homogenizer and centrifuged at 3000×g for 20 min at 4°C.

Oxidative stress biomarkers such as malondialdehyde (MDA), reduced glutathione (GSH), and reactive oxygen species (ROS) were assessed on the supernatant of the liver homogenate.

### 2.4. Biochemical Assays

The serum levels of aspartate aminotransferase (AST) and alanine aminotransferase (ALT) were tested with a biochemical autoanalyzer using commercially available kits (Nanjing Jiancheng Bioeng Inst, China) according to the manufacturer's instructions. Serum ALT and AST were expressed as U/ml.

Hepatic glycogen content was measured using mouse liver glycogen ELISA assay kit (Abcam, Cambridge, UK) according to the manufacturer's instructions. The samples from 10 mice in each group and the standard curves run in duplicate. The standard curves were obtained from standard samples, ranged from 0.6 to 9.6 mg/ml. The absorbance of glycogen standards and samples was recorded by a microplate reader at 450 nm (Bio-Tek Elx800, Bio-Tek), and the results were expressed as mg/ml. The coefficient of intra-assay variation was calculated as SD/mean × 100%.

### 2.5. Histopathological Examination

The liver samples were fixed in 10% phosphate-buffered formaldehyde for 48 h. After fixation, the specimens were dehydrated with graded ethanol, cleared in xylene, and embedded in paraffin wax. Blocks were made and sectioned at a thickness of 4 *μ*m. The sections were deparaffinized, washed with PBS, and stained with hematoxylin and eosin (H&E). The stained slides were examined under a light microscope (IX 70, Olympus, Tokyo, Japan).

### 2.6. Ultrastructure Analysis by Transmission Electron Microscopy

The liver samples were fixed in chilled 4% glutaraldehyde for 48 h and postfixed in 1% osmium tetroxide in water for one hour. After dehydrating with ethanol, the samples were infiltrated with acetone-araldite, embedded in araldite. Then, ultrathin sections were cut and stained with uranyl acetate and lead citrate. Labeled ultrathin sections were observed with a transmission electron microscope (JEM-2100;Joel Electron Inc., Tokyo, Japan).

### 2.7. Measurement of MDA Content, Production of ROS, Reduced GSH Level in Liver Homogenates, and AOPPs in Serum

Malondialdehyde (MDA) is a breakdown product of lipid peroxidation. MDA level in the liver was determined using a thiobarbituric acid reactive species (TBARS) assay kit (Nanjing Jiancheng Bioeng Inst, China) according to the manufacturer's instructions. The 10% liver homogenates prepared as above were incubated with TBA color reagents at 50°C for 60 min. The absorbance of red TBA-MDA cocktail was measured with a microplate reader at 532 nm (Bio-Tek Elx800, Bio-Tek), and the results were presented as nmol/ml.

Intracellular ROS production was measured using ROS-sensitive probe 2′,7′-dichlorodihydrofluorescein diacetate (DCFH-DA, Molecular Probes, Eugene, OR). The 10% homogenates were diluted 1 : 2 and loaded with DCFH-DA (5 *μ*M) at 37°C in the dark for 30 min. Thereafter, the samples were washed twice with PBS to remove the excess probe. The fluorescent product DCF was measured by fluorescence spectrophotometer (F-7000, Hitachi, Japan) with excitation at 485 nm and emission at 530 nm.

The level of advanced oxidative protein products (AOPPs) in the serum was measured using the specific ELISA kit (QiMing Biotechnology Co. Ltd., Shanghai, China) according to the manufacturer's instructions. The samples from six mice in each group and the standard curves run in duplicate. The standard curves were obtained from standard samples, ranged from 30 to 480 pmol/l. The absorbance was measured at 450 nm with a microplate reader (Bio-Tek Elx800, Bio-Tek). The coefficient of intra-assay variation was calculated as SD/mean × 100%.

The level of reduced glutathione (GSH) is reduced in the presence of oxidative stress. It was determined by a commercially GSH assay kit (Nanjing Jiancheng Bioeng Inst, China) according to the manufacturer's instructions. The concentrations of GSH were expressed as nmol/mg protein.

### 2.8. TNF-*α* Level in Liver Homogenates

The TNF-*α* level in the liver homogenates was measured with a specific ELISA kit (QiMing Biotechnology Co. Ltd., Shanghai, China). The samples were diluted 1 : 5. The samples, the standards, and the blank were run in duplicate. The standard curves were obtained from standard samples, ranged from 25 to 400 pg/ml. The absorbance was measured at 340 nm with a microplate reader (Bio-Tek Elx800, Bio-Tek). The coefficient of intra-assay variation was calculated as SD/mean × 100%.

### 2.9. Total RNA Isolation and Quantitative Real-Time PCR

To determine the mRNA expression level of inflammation cytokines (TNF-*α*, IL-6, IL-1*β*, and NF-*κ*B) in the liver, total RNA was extracted from frozen liver samples using TRIzol reagent according to the manufacturer's instructions and the purity of RNA was confirmed using measures of absorbance at 260 and 280 nm. From 1 *μ*g of resulting total RNA, cDNA products were synthesized using reverse transcriptase kits (Promega Corp., Madison, WI) and synthesized cDNA was amplified by FastStart Universal SYBR Green master mix (TaKaRa Biotechnology, Dalian, China) in a total volume of 20 *μ*l using the primer set described in Table 1. PCR cycles included initial denaturation at 95°C for 5 min and 40 cycles of denaturation at 95°C for 15 s, annealing at Tm-5 for 60 s and extension at 72°C for 30 s. The reactions were conducted on the ABI PRISM 7500 Real-time PCR system (Applied Biosystems, USA). Samples were run in triplicate with RNA preparations from three independent experiments. The relative mRNA expression level was calculated using the comparative 2^-*△△*CT^ method, and the values were normalized to *β*-actin.

### 2.10. Western Blotting Analysis

Liver tissues were lysed in ice-cold RIPA lysis buffer (Beyotime Biotechnology, Shanghai, China) containing protease inhibitors and phosphatase inhibitors and then centrifuged at 12000 g for 15 min at 4°C and collected the supernatants carefully. Protein concentrations were determined by BCA protein assay kit (Beyotime Biotechnology, China). Equivalent amounts of protein were separated by 10% SDS polyacrylamide gel electrophoresis and transferred to PVDF membranes. The membranes were blocked in 5% (*w*/*v*) nonfat milk powder in TBST (Tris-buffered saline with 0.1% Tween) for 1 h at room temperature. The membranes were incubated with rabbit primary antibodies for TLR4, NF-*κ*B, MYD88, TRAF6, P38, p-P38, JNK1/2, p-JNK1/2, and *β*-actin from ImmunoWay Biotechnology Company (Plano, TX, USA), respectively, diluted in blocking buffer at 4°C overnight. After washing with TBST for 3 times, the membranes were incubated with peroxidase-conjugated anti-rabbit secondary antibody (1 : 5000, Abcam USA) for 1 h at room temperature and then washed with TBST for 3 times. The immunoblots were developed using a chemiluminescence kit (Beyotime Biotechnology, China) and visualized by a chemiluminescence system (Tanon 5200). The band intensity was quantified using ImageJ, normalized to *β*-actin.

### 2.11. Statistical Analysis

All data were reported as means ± SE (standard error). Statistical analysis was performed with SPSS 21.0 software (Chicago, IL, USA). All statistical comparisons were made by means of the one-way ANOVA test followed by LSD post hoc analysis. *P* values < 0.05 were considered as statistically significant and *P* values of <0.01were considered highly significant.

## 3. Results

### 3.1. Changes in Body Weight and Liver Weight after DBA oral Exposure

**Figure 1:**
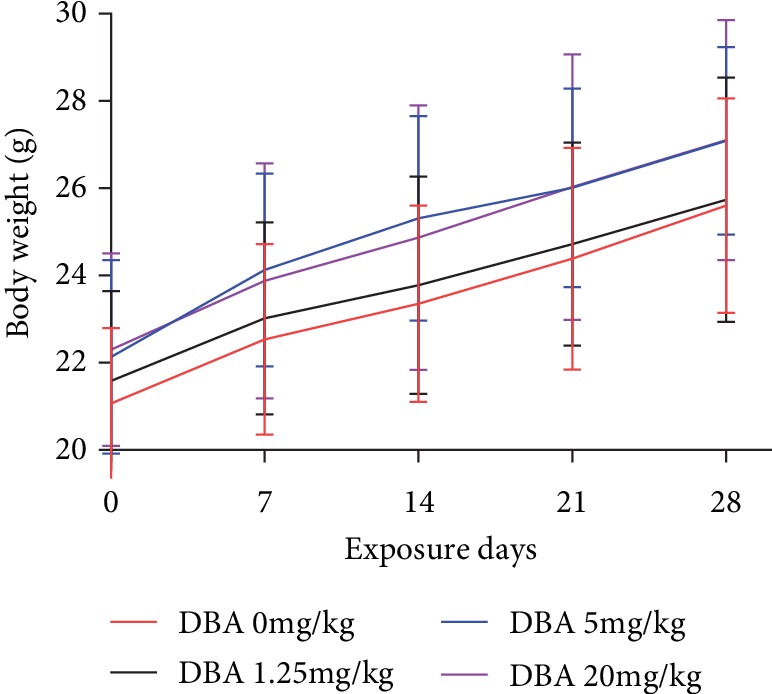
The changes in body weight of Balb/c mice after oral exposure to DBA each week. The values represent mean ± standard error (SE), *n* = 10.

All animals survived until the end of the study. There were no overt changes in appearance and/or behavior following DBA oral exposure. All animals gained weight, and no significant differences in body weight were observed. The mean liver weight increased in the 5 mg/kg and 20 mg/kg dose group and its relative weight increased in all dose groups (as shown in Figure 1 and Table 2).

### 3.2. Effects of DBA on Liver Function Markers

**Figure 2:**
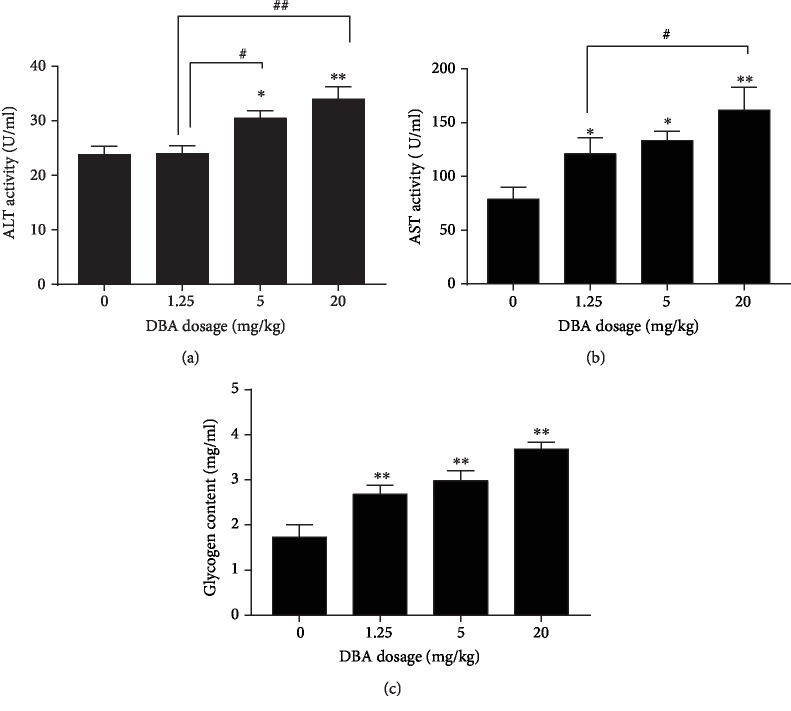
The serum levels of alanine aminotransferase (ALT), aspartate aminotransferase (AST), and glycogen. Balb/c mice were orally administrated with DBA at the dose of 1.25, 5, and 20 mg/kg for 28 days. After DBA treatment, the mice were sacrificed. Serum was separated from whole blood and analyzed for (a) ALT, (b) AST, and (c) glycogen. The values represent mean ± standard error (SE), *n* = 10. ^∗^*P* < 0.05,^∗∗^*P* < 0.01 versus the control group. ^#^*P* < 0.05, ^##^*P* < 0.01 represent statistically significant difference between the two dose groups.

Circulating levels of the liver enzymes ALT and AST were determined as indices of liver damage. The effects of DBA-induced hepatotoxicity on serum markers of liver function are shown in Figure 2(a) and 2(b). Serum activities of the liver AST and ALT were significantly increased in DBA-administered mice in comparison with the control mice. Additionally, DBA treatment induced glycogen accumulation in the liver of mice, and the data showed that liver glycogen was significantly higher in each dose group of DBA compared to the control (Figure 2(c)). The coefficient of intra-assay variation was 3.6%.

### 3.3. Alterations of Histopathological and Ultrastructure in the Liver of Balb/c Mice Caused by DBA

**Figure 3:**
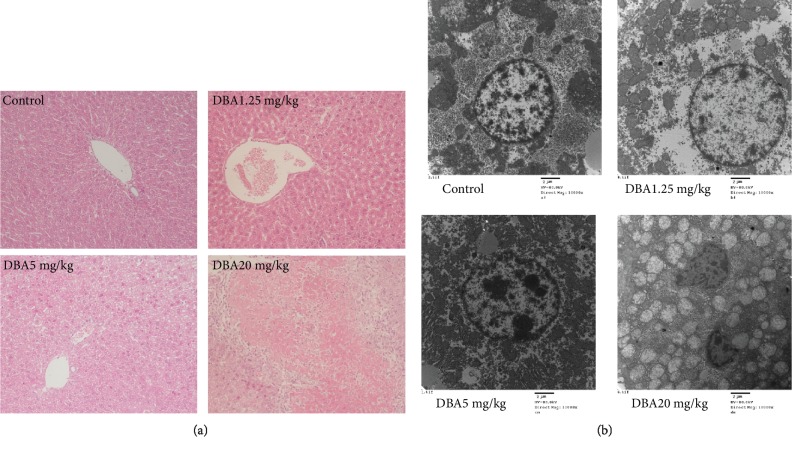
Oral administration of DBA induced obvious liver injury in histopathological changes. (a) Representative sections of formalin-fixed, paraffin-embedded livers stained with hematoxylin and eosin of each group are shown at ×100. (b) Representative transmission electron microscopy (TEM) of liver sections derived from the control or DBA-treated mice showed ultrastructure changes in mitochondrial and nucleus at ×10000.

Microscopic observation of H&E-stained liver section from the control mice showed normal histopathological appearance with the structural integrity of hepatic cells without necrosis or inflammation. Gross cellular damage was visible in the form of hepatic lesions, cytoplasmic vacuolation, and central vein dilatation in the DBA treatment groups. In the 20 mg/kg dose group, pyknotic body formation can be seen in the margin hepatic parenchyma as well as the focal cytolysis necrosis and local hemorrhage, as shown in Figure 3(a).

The subcellular alterations in hepatocytes induced by DBA were analyzed by transmission electron microscopy. It was confirmed that the mitochondria and the nuclei were indeed affected by DBA (Figure 3(b)). In the hepatocytes from control group mice, the nuclei were normal, with evenly distributed chromatin, and the mitochondria did not exhibit edema and had clear mitochondrial cristae. In contrast, hepatocytes from DBA-treated mice revealed that the mitochondria were swollen with no cristae, widespread nuclei shrinkage, chromatin karyopyknosis, and widened intranuclear space in a dose-dependent manner.

### 3.4. DBA Induced Changes in Oxidative Stress Biomarkers in the Liver

**Figure 4:**
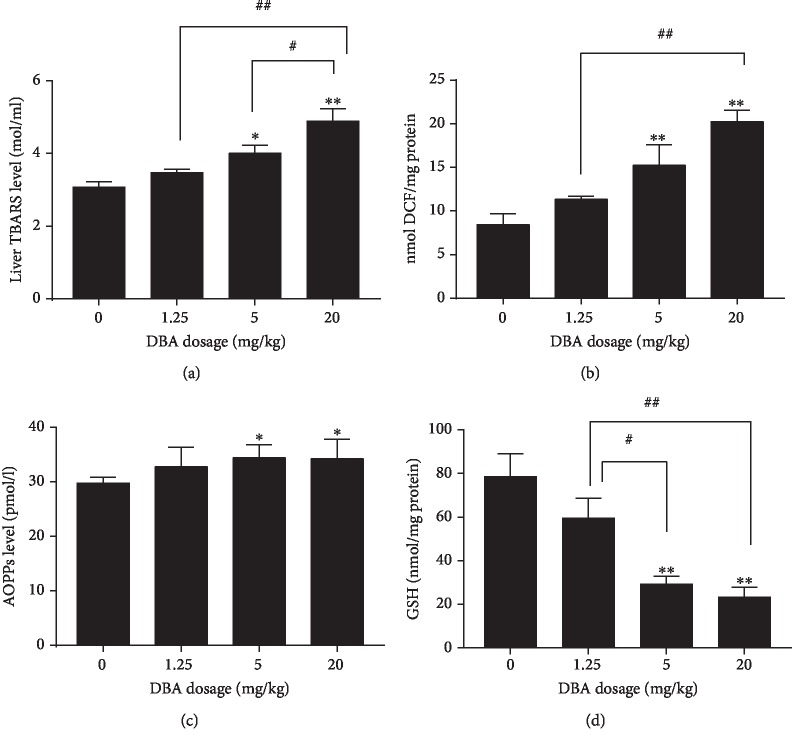
Oral administration of DBA induced oxidative stress and lipid peroxidation in the liver of the mice. (a) TBARS level in the liver. (b) ROS production in the liver. (c) AOPP levels in the serum. (d) GSH level in the liver of DBA administered mice. Data are mean ± SE, *n* = 6. ^∗^*P* < 0.05,^∗∗^*P* < 0.01 versus the control group. ^#^*P* < 0.05, ^##^*P* < 0.01 represent statistically significant difference between the two dose groups.

Oxidative stress was determined by measuring the levels of TBARS, ROS, AOPPs and GSH. TBARS levels were used as an index of lipid peroxidation in the liver. The data are presented in Figure 4(a). DBA administration induced a significant elevation of TBARS level in the 5 mg/kg and 20 mg/kg dose group in comparison with the control. The production of intracellular ROS in the livers in the 5 mg/kg and 20 mg/kg dose group increased compared with the control mice (Figure 4(b)). As shown in Figure 4(c), the level of AOPPs in the serum significantly increased in the 5 mg/kg and 20 mg/kg dose group compared with the control group. The coefficient of intra-assay variation was 8.1%.

In contrast, the level of the antioxidant enzyme GSH showed a significant decrease in the liver of the 5 mg/kg and 20 mg/kg dose group of DBA-administrated mice compared with the control group (Figure 4(d)). These findings indicated that oxidative stress may be a mechanism related to DBA-induced hepatotoxicity.

### 3.5. DBA Upregulated the Gene Expression of Proinflammatory Cytokines and NF-*κ*B

**Figure 5:**
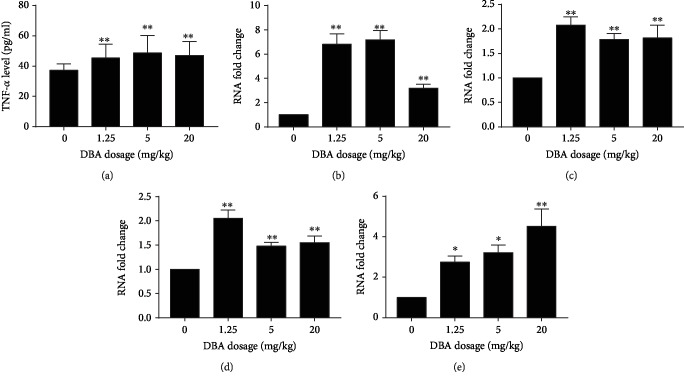
Oral administration of DBA increased the production of TNF-*α* and the levels of mRNA expression of TNF-*α*, IL-1*β*, IL-6, and NF-*κ*B in the livers of Balb/c mice. (a) The production of TNF-*α* was determined by ELISA assay. The values represent the mean ± SE, *n* = 10. The mRNA level of (b) TNF-*α*, (c) IL-1*β*, (d) IL-6, and (e) NF-*κ*B in the livers was analyzed by real-time quantitative polymerase chain reaction (qPCR). The values are shown as the fold change over the control group, *n* = 8. ^∗^*P* < 0.05,^∗∗^*P* < 0.01 versus the control group.

The inflammatory response was confirmed by upregulation of some proinflammatory cytokines in ELISA assay and qPCR analysis. As shown in Figures 5(a) and 5(b), both the production and mRNA expression of TNF-*α* in the liver significantly increased in all the DBA treatment group. The coefficient of intra-assay variation was 10.1% in the ELISA assay. In addition, the mRNA levels of IL-6 and IL-1*β* in the liver were also significantly increased induced by DBA, as shown in Figures 5(c) and 5(d). NF-*κ*B is an oxidant-sensitive transcription factor responsible for regulating inflammatory response gene expression. The data showed that DBA upregulated NF-*κ*B mRNA expression in the liver of the mice (Figure 5(e)).

### 3.6. DBA Activated TLR4 Signaling Pathway and MAPK Pathway

**Figure 6:**
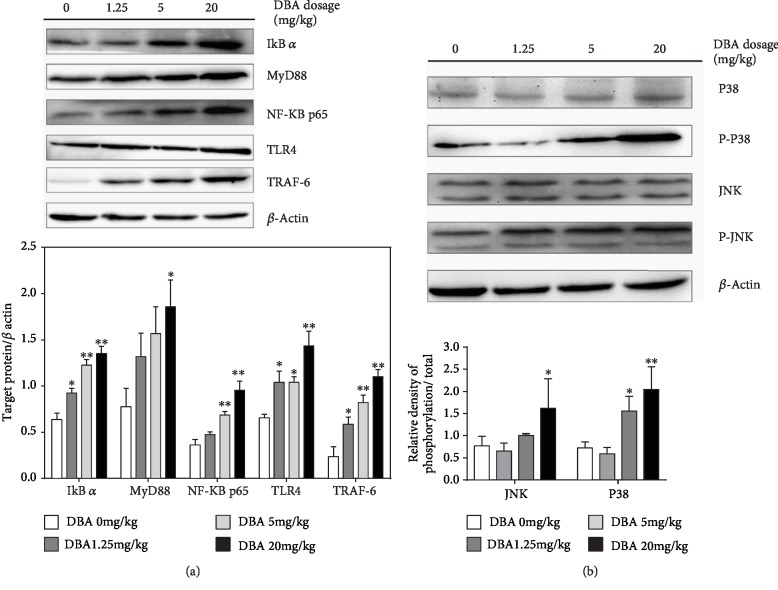
Effects of DBA on Toll-like receptor 4 (TLR4) and its adaptor molecules. Oral administration of DBA results in activation of TLR4-mediated MyD88-dependent pathway in the liver of Balb/c mice. (a) Representative blots for I*κ*B-*α*, MyD88, NF-*κ*B p65, TLR4, and TRAF-6 with the respective blot for *β*-actin used for normalization in the upper panel and the folder change of target protein/*β*-actin in each group in the bottom panel. (b) Representative blots for p38, phosphorylated p38, JNK, and phosphorylated JNK with the respective blot for *β*-actin used for normalization in the upper panel and the folder change of target protein/*β*-actin in each group in the bottom panel. The data are shown as mean ± SE, *n* = 3. ^∗^*P* < 0.05, ^∗∗^*P* < 0.01 versus the control group.

As shown in Figure 6(a), the protein levels of some key components in the TLR4 MyD88-dependent signaling pathway including MYD88, NF-*κ*B p65, I*κ*B-*α*, TLR-4, and TRAF6 were upregulated in the DBA-treated group, compared with the control group. Mitogen-activated protein kinase (MAPK) is expressed in all eukaryotic cells including P38 MAPK, ERK, and JNK subgroup. In the current study, P38 MAPK, JNK, and their phosphorylation levels were measured. The results showed that the phosphorylation level of P38 and JNK1/2 increased in the liver of the mice after DBA treatment, especially in 20mg/kg dose group (Figure 6(b)).

## 4. Discussion

Nowadays, liver injury caused by chemicals, a serious threat to people's health, is becoming more and more common. Few research works have been conducted to analyze the adverse effect of DBA on the liver. Therefore, the main aim of this study was to examine the hepatotoxicity of DBA and the underlying mechanisms. In the present study, the mice were administered with different doses of DBA by oral gavage for consecutive 28 days. After the treatment, although no obvious physical changes such as morbidity, mortality, or alterations in body weight were observed in any treatment group, higher liver weight and relative weight were observed.

The liver injury caused by DBA was confirmed by hepatic marker enzyme examination and histopathological examination. Elevated levels of serum hepatic marker enzymes such as ALT and AST indicated loss of functional integrity of cell membranes and cellular leakage when the liver cell plasma membrane is damaged [15, 16]. In the present study, the increased serum activity of AST and ALT confirmed that the extensive liver damage was induced by DBA, especially in the 5 mg/kg and 20 mg/kg dose group. Consistent with a previous study [17], DBA also induced glycogen accumulation in hepatocytes, which indicated that DBA might result in hepatic damage via disruption of glucose metabolism. Additionally, histopathological examination further confirmed this hepatic damage by hepatocellular necrosis, inflammatory cell infiltration, and central vein dilation and congestion. The higher magnification electron micrograph revealed swollen mitochondria and karyopyknosis in a dose-dependent manner.

We tried to investigate the underlying mechanisms of the hepatotoxicity of DBA. Reactive oxygen species (ROS) is normally produced in the mitochondrial electron transport chain (ETC) during respiration [18]. Excess ROS may damage cellular lipids, proteins, DNA, and active JNK, which ultimately evokes a large amount of cellular death and inflammation [19, 20]. When the production of ROS exceeds the capacity of cellular antioxidant machinery, imbalance in the prooxidant and antioxidant systems occurred leading to a state of oxidative stress and is also one contributor for liver injury [21, 22]. The most important indicators of oxidative stress are lipid peroxidation and protein oxidation, such as MDA, an end-product of lipid peroxidation products and AOPP, a new marker of protein oxidation [23–25]. The generation of ROS and lipid peroxidation products induced by DBA would have created damage to the hepatocyte membrane and results in the leakage of liver marker enzymes into the blood circulation [15, 26]. AOPPs are most notably albumin and its aggregates and high AOPPs levels are indicative of an increase in oxidative stress [27]. In some endogenous antioxidants such as GSH and CAT, the firstline of defense against oxidative damage played an important role in scavenging ROS, maintaining redox balances in the biological system and protecting the cells against lipid peroxidation [28]. In the present study, significant elevation of ROS, MDA in the liver and AOPPs in the serum, and decrease of GSH in the liver of DBA-treated mice were widely accepted signs of oxidative stress, especially in the 5 mg/kg and 20 mg/kg dose group.

ROS not only directly causes tissue damage but also initiates the inflammation [29]. Inflammation is one of the major mechanisms involved in liver injury initiated by TLR4 activation. TLR4, a crucial mediator in the activation of innate immune response, can activate NF-*κ*B by combining with MyD88, increase the degradation of I*κ*B-*α*, and thus promote the translocation of NF-*κ*Bp50 and p65 into nucleus, ultimately trigger signaling cascade with the release of proinflammatory cytokines including TNF-*α*, IL-1*β*, and IL-6, which can further aggravate the activation of NF-*κ*B signaling [30, 31]. The inflammatory cytokine TNF-*α* plays an important role in mediating hepatotoxic responses of some xenobiotics [32, 33]. The proinflammation factors including IL-6, TNF-*α*, and IL-1*β* have been shown to stimulate ROS accumulation, activate innate immunity, and lead to a secondary hit to hepatocytes, thus amplify the liver injury [7]. In our work, the production of TNF-*α* and the mRNA level of TNF-*α*, IL-1*β*, IL-6, and NF-*κ*B were significantly increased in hepatic tissues of Balb/c mice and also upregulated the protein levels of TLR4, MYD88, I*κ*B-*α*, TRAF-6, and NF-*κ*Bp65. This upregulation can directly lead to hepatocellular damage. These data demonstrated that DBA could induce inflammation and activation of the TLR4 pathway in the liver.

Upon TLR4 engagement, a receptor-associated complex containing MyD88, TRAF6, and transforming growth factor *β*-activated kinase 1 (TAK1) is formed. TAK1 is a crucial regulator in the TLR4 pathway which activates its downstream targets—NF-*κ*B pathway and MAPK cascades, such as p38, c-Jun N-terminal kinase (JNK), and extracellular signal-regulated kinase (ERK). TRAF6 interaction with TAK1 is responsible for the activation of the MAPK pathway [34]. MAPKs are also activated by diverse stimuli including mitogen, proinflammatory cytokines, and oxidative stress [35, 36]. The activation of MAPKs also stimulates the production of TNF-*α*, IL-6, and ROS which is also believed a major contributor to liver injury [37, 38]. In our study, the key proteins in MAPK family such as JNK and P38 were determined, and the results showed that DBA upregulated the protein levels of phosphorylated JNK and P38. The data suggested that P38 MAPK in addition to JNK participates in the liver injury induced by TLR4, ROS, or proinflammation cytokines. All the results suggested that DBA-induced liver injury may be through inflammation modulated by the MyD88-dependent TLR4 signaling pathway.

In summary, our study provides evidence that oral exposure to DBA for 28 days can induce liver injury in Balb/c mice; the mechanism is related to oxidative stress, inflammation, and TLR4/NF-*κ*B pathway activation. But the deep mechanisms on the liver injury caused by DBA needed further investigation.

## Figures and Tables

**Table 1:** The primers used for real-time RT-PCR.

Target gene	Sequence (5′-3′)
TNF-*α*	F: CCACGCTCTTCTGTCTACTA
	R: GTTTGTGAGTGTGAGGGTCTGCTAGA

IL-6	F: AACCACGGCCTTCCCTACT
	R: CATTTCCACGATTTCCCAGA

IL-1*β*	F: TTTGAAGTTGACGGACCCC
	R: GATGTGCTGCTGCGAGATT

*β*-Actin	F: CTATGCTCTCCCTCACGCC
	R: GCAACATAGCACAGCTTC

**Table 2:** The liver weight and liver relative weight to the body weight.

DBA dosage (mg/kg)	Liver weight (g)	Liver relative weight (%)
0	1.157 ± 0.16	4.487 ± 0.58
1.25	1.144 ± 0.12	4.646 ± 0.71
5	1.249 ± 0.14	4.808 ± 0.68
20	1.205 ± 0.14	4.654 ± 0.60

Note: animals were treated by oral gavage DBA for consecutive 28 days. Relative weight is liver weight/body weight∗100%. Data are presented as mean ± SE (*n* = 10 per group).

## Data Availability

The data used to support the findings of this study are available from the corresponding author upon request.

## Abbreviations

DBA: Dibromoacetic acid
NIEH: National Institute of Environmental Health Sciences
CYP 450: Cytochrome P450
NAPQI: N-acetyl-p-benzoquinone imine
GSH: Glutathione
TLRs: Toll-like receptors
PRRs: Pattern recognition receptors
MyD88: Myeloid differentiation factor 88
NF-*κ*B: Nuclear factor *κ*B
IKK: I*κ*B-*α* kinase
TNF-*α*: Tumor necrosis factor *α*
IL-6: Interleukin-6
IL-1*β*: Interleukin-1*β*
ALT: Alanine aminotransferase
AST: Aspartate aminotransferase
ROS: Reactive oxygen species
MDA: Malondialdehyde
TBARS: Thiobarbituric acid reactive species
DCFH-DA: 2′,7′-Dichlorodihydrofluorescein diacetate
AOPPs: Advanced oxidative protein products
TBST: Tris-buffered saline with 0.1% Tween
TRAF-6: TNF receptor-associated factor 6
TAK1: Transforming growth factor *β*-activated kinase 1
JNK: c-Jun N-terminal kinase
MAPKs: Mitogen-activated protein kinases.
